# Exploring the psychological factors influencing intention to use AI virtual companions: an SEM-ANN study

**DOI:** 10.3389/fpsyg.2026.1855345

**Published:** 2026-06-09

**Authors:** Yue Sun, Jun Liu, Ting Liu, Zhengqi Wei, Huajie Shen

**Affiliations:** 1 School of Design, Fujian University of Technology, Fuzhou, China; 2 College of Art, Cheongju University, Cheongju, Republic of Korea

**Keywords:** AI trust, artificial intelligence (AI), artificial neural network (ANN), structural equation modeling (SEM), virtual companion

## Abstract

**Introduction:**

AI virtual companion products have shown promise in providing emotional support and interactive experiences, but their user acceptance and broader adoption still face challenges. Building on the TAM3 model, this study introduced AI trust, perceived anthropomorphism, and social anxiety to construct a theoretical model of usage intention.

**Methods:**

Using survey data from 712 users in China, the study employed both PLS-SEM and ANN for empirical analysis.

**Results:**

The results showed that perceived usefulness, perceived enjoyment, AI trust, and perceived anthropomorphism were all significantly and positively associated with usage intention, whereas the direct associations of perceived ease of use and social anxiety with usage intention were not significant. In addition, gender showed significant differences in the paths linking AI trust and perceived enjoyment to usage intention, while age showed significant differences in the paths linking AI trust and perceived anthropomorphism to usage intention. Both SEM and ANN indicated that AI trust was the most critical predictor, whereas perceived usefulness had the lowest relative importance. However, the two methods differed in their ranking of the intermediate predictors: in SEM, perceived enjoyment was more important than perceived anthropomorphism, whereas the opposite pattern emerged in ANN.

**Discussion:**

The originality of this study lies in its theoretical extension of the user acceptance framework, its methodological demonstration of the advantages of integrating SEM and ANN, and its practical implications for optimizing virtual companion products. Specifically, the findings suggest the need to balance technical safety with emotional experience and to adopt differentiated design and promotion strategies for different user groups.

## 1 Introduction

A virtual companion refers to a digital entity capable of interacting with humans and providing functions such as emotional support and social interaction, originally designed to offer companionship for users with needs for emotional comfort and social substitution (Strohmann et al., 2023). In modern society, factors such as migration, the accelerating pace of urban life, and the pursuit of independent living have led to a growing number of individuals living alone, making the issue of companionship deficiency increasingly prominent (Berridge et al., 2023; Gasteiger et al., 2022; Rook, 1987).

Against this backdrop, virtual companion products have emerged, designed as emotional interaction systems to respond to users' affective needs (Picard and Klein, 2002). Common examples include virtual assistant products, embodied conversational agents, chatbots, and virtual humans (Boumans et al., 2022). Research has shown that such companion-oriented products hold potential for alleviating negative emotions such as anxiety, loneliness, and stress (Odekerken-Schröder et al., 2020). However, early studies on virtual companions primarily relied on rule-based dialogue systems, such as Eliza (Weizenbaum, 1966), Parry (Colby et al., 1971), and Alice (Wallace, 2009), which depended on keyword matching and fixed templates. These systems were only capable of simple conversations in limited contexts and struggled to achieve natural and fluent communication (Zhou et al., 2020). At the same time, scholars have pointed out that current virtual companion products still face technical bottlenecks in deep emotion recognition and nuanced empathetic feedback, which restrict their effectiveness and credibility in emotional support scenarios (Cui and Liu, 2023).

With the continuous advancement of artificial intelligence (AI) technologies, AI has been widely applied in fields such as education, psychological counseling, health management, and entertainment (Dogan et al., 2023; Liu, 2024; Oke, 2008; Wang and Luo, 2025; Wang and Xiao, 2025). Among these applications, AI virtual companion products represent a category of interactive intelligent systems powered by natural language processing (NLP) and machine learning technologies, which can generate responses based on user needs and are commonly used in contexts of intelligent dialogue and emotional communication (Schuetzler et al., 2020; Shawar and Atwell, 2007). These systems hold significant value in affective interaction research and have gradually become a focal point for both academic inquiry and industrial development (Chaturvedi et al., 2023). Studies have demonstrated that AI virtual companion products, through emotion recognition, personalized responses, and empathetic interactions, can effectively meet users' emotional needs and alleviate loneliness, thereby fostering deep trust (Kouros and Papa, 2024). Other research has further suggested that AI virtual companions not only exhibit strong interactive capabilities in emotional companionship but also possess the potential to approach or even surpass human experts in areas such as cognitive understanding, language generation, and human-computer communication (Chou et al., 2025). At the same time, global technology companies are increasingly prioritizing the development and application of AI virtual companion products (Strohmann et al., 2023). For example, Replika and Microsoft's XiaoIce have become representative commercial success stories: Replika has attracted over 7 million users, while XiaoIce boasts more than 660 million active users, highlighting the tremendous global market potential of AI virtual companions (Zhou et al., 2020). Looking ahead, AI virtual companion products are expected to see broader applications in both daily work and everyday life (Chan and Chou, 1997).

Although scholarly attention to AI virtual companion products has been steadily increasing (Mogaji et al., 2021), existing studies have not sufficiently explained their impact on users' emotional cognition and interactive experiences, particularly lacking a systematic analysis of user acceptance (Kefi et al., 2024). Unlike general task-oriented intelligent assistants or functional chatbots, the core value of AI virtual companion products lies not only in information response and functional support, but also in emotional companionship, parasocial interaction, personalized relationship building, and sustained interactive experiences. Therefore, relying solely on traditional technology acceptance variables is insufficient to fully explain users' intention to use this type of emotionally relational AI product. Against this background, the present study builds on an extended TAM3 framework and introduces variables such as AI trust, perceived anthropomorphism, and social anxiety to construct a model of users' usage intention. It should be emphasized that the contribution of this study does not lie in simply combining existing variables, but in integrating traditional technology acceptance factors, AI relational-feature factors, and social-psychological factors within the emotionally relational technological context of AI virtual companions. Methodologically, the study adopts a hybrid approach that combines structural equation modeling (SEM) and artificial neural networks (ANN) to test the linear association paths in the theoretical model and to further identify the relative importance of significant predictors within a nonlinear predictive framework. This study not only helps extend the theoretical framework for explaining user acceptance of AI virtual companion products, but also provides practical implications for trust-building mechanisms, anthropomorphic interaction design, and differentiated user engagement strategies.

## 2 Literature review and hypothesis development

### 2.1 Theoretical foundation

The Technology Acceptance Model (TAM), proposed by Fred Davis, was developed to explain and predict users' acceptance and usage behavior toward new technologies or information systems (Venkatesh and Davis, 1996). The Technology Acceptance Model 3 (TAM3) is an extended and refined version of the original TAM and has become a mainstream framework for analyzing the factors influencing users' acceptance and use of technology (Faqih and Jaradat, 2015). As an emerging form of human-computer interaction, AI virtual companion products are well-suited for investigation through TAM3 to explore users' behavioral intentions and their underlying determinants. Accordingly, this study adopts TAM3 as the theoretical foundation to construct a model of factors influencing the usage intention of AI virtual companion products.

Existing TAM3-based studies have provided empirical evidence that cognitive and affective factors such as perceived usefulness (PU), perceived ease of use (PEU), and perceived enjoyment (PE) can significantly enhance users' usage intentions (Algerafi et al., 2023). However, some scholars have criticized the TAM framework for overemphasizing external motivational variables (e.g., PU and PEU) while neglecting intrinsic user motivations (Taherdoost, 2018). Moreover, most TAM-related studies have insufficiently considered users' negative perceptions, thereby limiting the model's explanatory power regarding actual usage behavior (Al-Adwan et al., 2023). To address these limitations, researchers have attempted to introduce extended variables—such as affective perceptions and individual characteristics—to improve the model's applicability (Kefi et al., 2024; Kuncoro et al., 2019).

Recent studies have shown that, in AI-related product contexts, product-related psychological perceptions such as AI trust and perceived anthropomorphism, as well as user-specific psychological characteristics such as social anxiety, may all be closely related to the technology acceptance process (Hsieh and Lee, 2021; Hu et al., 2023; Zhang et al., 2021). Among these factors, AI trust and perceived anthropomorphism mainly reflect users' psychological perceptions of AI virtual companion products, whereas social anxiety reflects users' own individual psychological characteristics. Given that AI virtual companions possess emotionally relational attributes such as emotional companionship, human-like interaction, relational continuity, and privacy-related trust, this study does not simply combine existing variables, but instead extends TAM3 based on the specific product context. Accordingly, this study incorporates AI trust, perceived anthropomorphism, and social anxiety into an extended TAM3 model to enhance its explanatory power for users' intention to use AI virtual companion products. At the same time, gender and age are included as moderating variables to examine their moderating effects on behavioral intention. This model serves as the theoretical foundation for the subsequent structural equation modeling (SEM) and artificial neural network (ANN) analyses. Accordingly, this study incorporates AI trust, perceived anthropomorphism, and social anxiety into an extended TAM3 model to enhance its explanatory power for users' intention to use AI virtual companion products. At the same time, gender and age are included as moderating variables to examine their moderating effects on behavioral intention. This model serves as the theoretical foundation for the subsequent structural equation modeling (SEM) and artificial neural network (ANN) analyses.

### 2.2 Research hypotheses

#### 2.2.1 Perceived usefulness

Perceived usefulness refers to the degree to which users believe a technological system is valuable in improving life efficiency or providing practical assistance (Wang et al., 2025). In the context of AI virtual companion products, perceived usefulness primarily reflects users' evaluations of whether the system offers tangible benefits in areas such as daily life assistance, emotional companionship, and psychological support (Kim et al., 2021). Studies have shown that in elderly care, AI virtual companions enhance users' independence and improve their life satisfaction, thereby increasing their perceived usefulness in real-world applications (Jegundo et al., 2020). Furthermore, research indicates that when users believe AI virtual companions can alleviate loneliness and enhance wellbeing, their perception of usefulness significantly increases, which in turn promotes usage intention (Merrill Jr et al., 2022). Based on this, the following hypothesis is proposed:

**H1:** Perceived usefulness is positively associated with usage intention.

#### 2.2.2 Perceived ease of use

Perceived ease of use refers to users' subjective perception of whether a technological system is easy to learn and operate during use (Gefen and Straub, 2000). When users perceive a system as more user-friendly and easier to operate, their willingness to adopt the technology typically increases (Davis, 1989). In the context of AI virtual companion products, perceived ease of use can reduce learning costs and operational difficulties, thereby enhancing the fluency of human-computer interactions in daily tasks and ultimately increasing overall usage intention (Alshammari and Babu, 2025). Research has shown that users' acceptance of AI virtual companion products is significantly influenced by perceived ease of use, perceived usefulness, and perceived enjoyment, among which perceived ease of use is one of the key factors driving positive usage attitudes (He et al., 2023). Furthermore, other studies have indicated that in contexts such as AI virtual companions, perceived ease of use not only directly affects users' usage intention but also indirectly facilitates their technology adoption behavior (Liu et al., 2023). Based on this, the following hypothesis is proposed:

**H2:** Perceived ease of use is positively associated with usage intention.

#### 2.2.3 Perceived enjoyment

Perceived enjoyment refers to the psychological state of pleasure experienced by users when engaging with a technology, which is closely associated with emotional and cognitive factors (Hong and Tai, 2025). In the context of AI virtual companion products, enjoyment is derived not only from functional performance but also from the emotional release and entertainment satisfaction generated during interactions (Shao and Kwon, 2021). Studies have shown that AI virtual companions can enhance users' enjoyment and sense of immersion through personalized and emotionally engaging interactions, such as voice communication, contextual responses, and content recommendations, thereby strengthening their intention to use the system (Hsieh and Lee, 2021). Other research has further indicated that perceived enjoyment is a key psychological driver of continued usage of AI virtual assistants; when users perceive interactions with AI virtual assistants as more interesting, their acceptance and usage intention increase significantly (Jo, 2022). Based on this, the following hypothesis is proposed:

**H3:** Perceived enjoyment is positively associated with usage intention.

#### 2.2.4 AI trust

AI trust refers to users' positive expectations regarding the capability, reliability, and emotional sincerity of an artificial intelligence system (Bach et al., 2024). Studies have shown that when users develop trust in a system, they are more inclined to accept and adopt the technological services it provides (Almaiah et al., 2020; Ghosh et al., 2021). Research based on the TAM framework has further demonstrated that AI trust exerts a significant influence on users' intention to use AI systems, with trust in their technical competence and reliability playing an especially prominent role. This underscores the critical importance of building trustworthy AI systems to enhance user acceptance (Choung et al., 2023). Additional studies focusing on AI virtual assistants have also confirmed the central role of AI trust in promoting usage intention and improving user acceptance (Zhang et al., 2021). Based on this, the following hypothesis is proposed:

**H4:** AI trust is positively associated with usage intention.

#### 2.2.5 Perceived anthropomorphism

Perceived anthropomorphism refers to the extent to which users attribute human-like traits—such as tone, behavior, and emotions—to non-human technological objects (Bartneck et al., 2009). With advances in machine learning, natural language processing, and voice and image recognition, artificial intelligence systems have gradually acquired the ability to simulate human intelligent behaviors and display varying degrees of anthropomorphic features (Alabed et al., 2022). In the context of AI virtual companions, perceived anthropomorphism is reflected not only in human-like appearance or behavior, but also in users' perceptions of human-like mental attributes formed through language style, emotional responses, and interaction patterns. Research has shown that when AI virtual companions demonstrate human-like attributes such as language expression, interactive capability, and social roles, users are more likely to develop parasocial interactions. This, in turn, enhances trust and a sense of intimacy, ultimately increasing their acceptance of and willingness to continue using the system (Hsieh and Lee, 2021). Further studies on AI voice assistants have confirmed that perceived anthropomorphism significantly promotes users' intention to continue using the technology, as users often rely on human-like characteristics to evaluate system credibility (Zhou et al., 2023). Based on this, the following hypothesis is proposed:

**H5:** Perceived anthropomorphism is positively associated with usage intention.

#### 2.2.6 Social anxiety

Social anxiety is characterized by fear of social situations and interpersonal interactions, which can have profound effects on an individual's quality of life (Weeks et al., 2008). Research suggests that because AI virtual companions are not human entities that impose real social evaluation on users, individuals may experience less pressure from others' judgments when interacting with them. People with higher levels of social anxiety may therefore be more inclined to communicate with systems rather than with other people (Suzuki et al., 2022). Other research has further indicated that AI virtual companions provide open and safe channels for emotional communication, enabling users to express their feelings without fear of judgment, thereby reducing social anxiety to some extent and enhancing their willingness to accept technological services (Xie and Wang, 2024). Moreover, studies have found that users with higher levels of social anxiety often avoid interpersonal interactions due to feelings of embarrassment or discomfort in social contexts, making them more prone to frequent use of AI virtual companions and, in some cases, even overdependence (Kim et al., 2021). Based on this, the following hypothesis is proposed:

**H6:** Social anxiety is positively associated with usage intention.

#### 2.2.7 Moderating variables

Considering that user characteristics may moderate the relationship between influencing factors and usage intention, this study selects gender and age as moderating variables. Prior research has shown that individual characteristics can play a moderating role in the relationship between influencing factors and behavioral intention (Hamakhan, 2020). For example, one study indicated that perceived enjoyment is a key driver of users' acceptance and continued use of AI assistants, and its effect on behavioral intention differs across gender groups (Jo, 2022). Related research further found that perceived enjoyment significantly affects preservice teachers' willingness to use AI systems, with gender serving as a moderator in this relationship (Zhang et al., 2023). Gender differences have also been observed in trust: male users generally exhibit higher levels of AI trust, which further enhances their intention to use such systems (Kozak and Fel, 2024).

With respect to age, research has revealed that younger users tend to place greater trust in AI systems, making them more willing to use these products, whereas older users show relatively lower levels of trust, thereby reducing their usage intention (Symasek et al., 2025). In addition, younger users are more likely to strengthen their usage intention due to anthropomorphic features, while older users tend to adopt a more cautious attitude (Symasek et al., 2025).

Based on these findings, the following hypotheses are proposed:

**H7:** Gender moderates the relationships between perceived enjoyment, AI trust, and usage intention.
**H8:** Age moderates the relationships between AI trust, perceived anthropomorphism, and usage intention.

**Figure 1:**
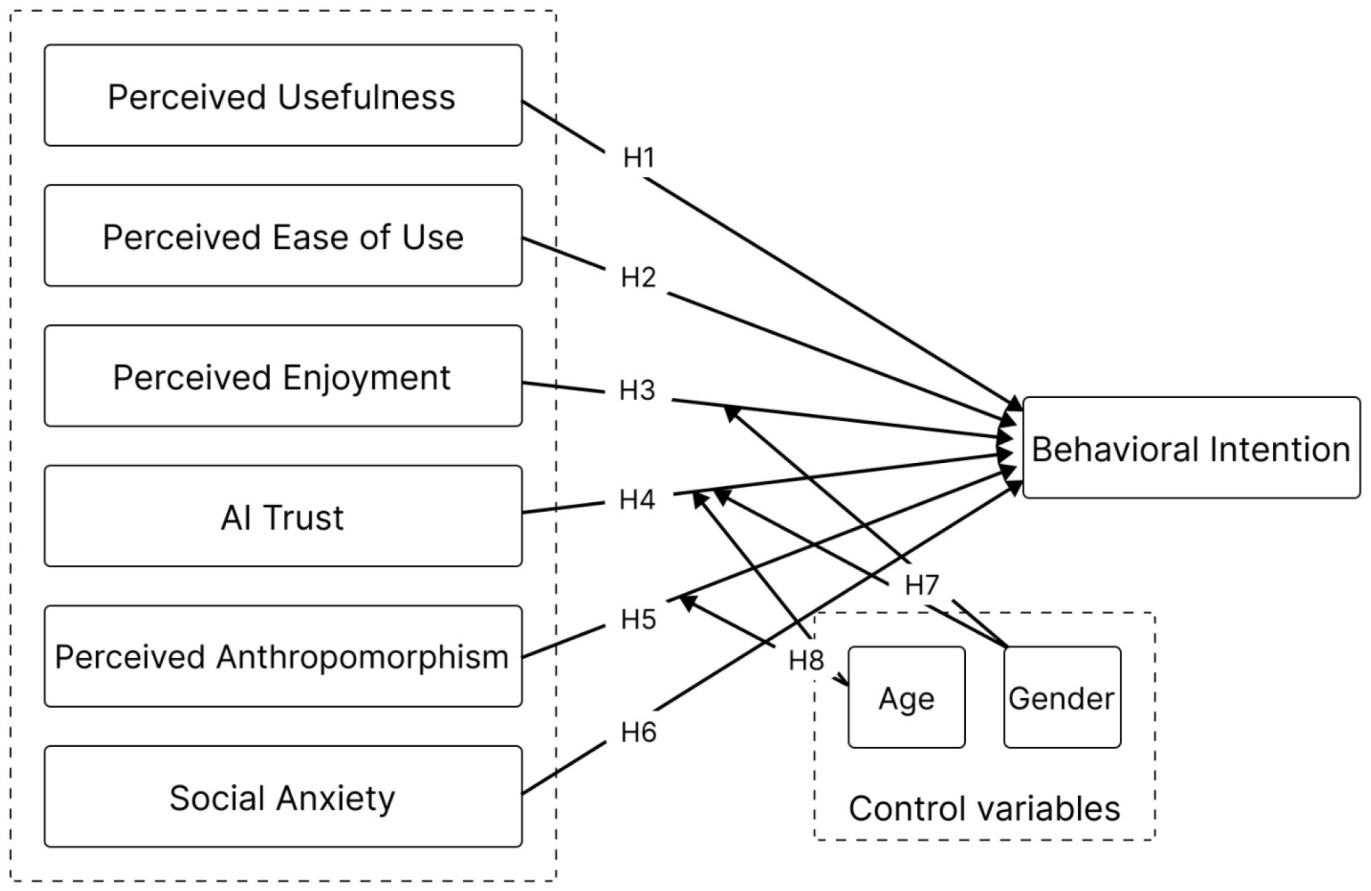
Conceptual model.

In summary, this study incorporates three fundamental variables—perceived usefulness (PU), perceived ease of use (PEU), and perceived enjoyment (PE)—along with three extended variables—AI trust (AIT), perceived anthropomorphism (PA), and social anxiety (SA). Gender and age are included as moderating variables to construct a conceptual model of users' intention to use AI virtual companion products, as shown in Figure 1.

## 3 Research design

### 3.1 Research method

Based on the conceptual model described above, this study employed Partial Least Squares Structural Equation Modeling (PLS-SEM) to test the research model and hypotheses in order to examine the factors associated with users' intention to use AI virtual companion products and their relative predictive effects. PLS-SEM allows researchers to test models containing multiple latent variables and their interrelationships, providing a rigorous approach for hypothesis testing and model evaluation (Jain et al., 2023). However, as a variance-based methodological framework, PLS-SEM generally assumes linear relationships among latent variables, and this assumption may not fully capture the complexity of user behavior (Bellary et al., 2024). Prior studies have suggested that Artificial Neural Networks (ANN) can serve as a complementary predictive method to SEM by identifying potential nonlinear relationships between input variables and outcome variables and by assessing the relative importance of significant predictors (Xu et al., 2023).

Accordingly, the SEM-ANN hybrid approach adopted in this study was not intended as a simple repetition of analysis, but rather was based on the complementarity of the two methods. PLS-SEM was mainly used to test the linear association paths in the theoretical model and their significance, whereas ANN was used to further examine the relative contribution of the significant variables identified in SEM to usage intention within a nonlinear predictive framework. Because ANN has a certain “black box” nature and cannot be used for path significance testing or theory-driven hypothesis testing in the same way as SEM, this study treated ANN as a supplementary predictive analysis tool following SEM rather than as a method replacing SEM.

The analytical procedure of this study consisted of three steps. First, SPSS was used to conduct descriptive statistical analysis in order to clarify the basic characteristics of the sample. Second, SmartPLS 3.0 was used to test the reliability and validity of the measurement model and to evaluate the association paths among latent variables in the structural model. Finally, based on the PLS-SEM results, predictors that reached statistical significance were entered into the ANN model, and the neural network module in SPSS was used for analysis to assess the relative predictive importance of each variable for intention to use AI virtual companion products.

### 3.2 Sample selection and data collection

Prior to the formal study, a small-scale pilot test was conducted to verify the reliability and validity of the questionnaire. The questionnaire was designed using the “Wenjuanxing” platform and employed a 7-point Likert scale. To ensure respondents fully understood the rating criteria, the scale was reiterated at the beginning of the survey, ranging from 1 (“strongly disagree”) to 7 (“strongly agree”). An informed consent statement was presented on the first page of the questionnaire, and only participants who confirmed their consent were allowed to proceed.

To ensure that respondents had a relatively consistent understanding of the research object, this study provided a unified explanation of “AI virtual companion products” before the formal questionnaire items. In the questionnaire, AI virtual companion products were defined as AI-based software products on smartphones or computers that provide functions such as chatting, interaction, and emotional response, and that can accompany users in an anthropomorphic way. To help respondents understand the research object, the questionnaire listed several common products, including Xingye, Serenade, Maopaoya, Maoxiang, Wow, Zhumengdao, Zaomeng Ciyuan, and X EVA, as representative examples, while also clarifying that the research object included but was not limited to these products. Respondents were allowed to continue only if they had used the above products or AI virtual companion products with similar functions. Therefore, this study focused on the overall intention to use this category of products among users with experience using AI virtual companion products, rather than on evaluations of any single platform or application.

During the data cleaning phase, incomplete or abnormal responses were removed, and the following criteria were applied: all required items had to be completed, and in the case of duplicate submissions from the same IP address or device, only the first valid response was retained. The questionnaire was distributed through WeChat and related groups, resulting in 40 valid pilot responses. Reliability testing was conducted using Cronbach's α to assess the internal consistency of each measurement dimension, with results showing all α values above 0.7, indicating good reliability (Izah et al., 2023). Construct validity was verified through Exploratory Factor Analysis (EFA), with KMO values exceeding 0.8 and Bartlett's test of sphericity showing significance (*p* < 0.001). Factor loadings were clear, confirming satisfactory convergent and discriminant validity of the scale (Eisinga et al., 2013; Tian and Hong, 2012). These results indicated that the questionnaire design was reasonable and capable of effectively capturing the meaning of the latent constructions.

On this basis, the study proceeded to the large-scale data collection stage, and a total of 776 questionnaires were ultimately collected. According to the questionnaire instructions, respondents were required to have experience using AI virtual companion products or products with similar functions before they could continue. After removing incomplete questionnaires and invalid responses with identical answers throughout, 712 valid questionnaires were retained, yielding an effective response rate of 91.8%. The sample size exceeded 10 times the number of measurement items, meeting the sample size requirement for PLS-SEM analysis (Liu and Sun, 2025).

### 3.3 Variable measurement

To ensure the validity of the measurement scales, the questionnaire design was informed by prior research. First, established scales from previous studies were adopted to ensure that the measurement dimensions and items had a solid theoretical foundation. Then, some items were appropriately revised to better fit the research context of AI virtual companion products. Among them, the perceived anthropomorphism items were adapted from existing perceived anthropomorphism scales developed in the contexts of AI assistants and AI systems, with a focus on measuring the human-like perceptions users form during interaction with AI virtual companions. After expert review and pilot testing, the final formal questionnaire was established, containing 21 measurement items. The specific variables and their corresponding measurement items are presented in Table 1.

**Table 1:** Questionnaire dimensions and specific content.

Construct	Measure	Sources
Perceived usefulness	I believe that AI virtual companion products meet my needs.	Fan and Jiang, 2024; Jegundo et al., 2020; Wang et al., 2025; Zhang et al., 2025
	I believe that using AI virtual companion products can improve my quality of life.	
	Overall, I believe that AI virtual companion products are very helpful to me.	
Perceived ease of use	I find AI virtual companion products easy to operate.	Jegundo et al., 2020; Venkatesh and Davis, 2000; Wang et al., 2025
	I find it effortless to learn how to use AI virtual companion products.	
	I believe that AI virtual companion products are user-friendly.	
AI trust	I believe that AI virtual companion products will not leak my personal information.	Choung et al., 2023; Gefen et al., 2003; Jin and Youn, 2021; Saleh et al., 2022
	I believe that AI virtual companion products have adequate security measures that make me feel safe using their services.	
	I am confident in the reliability of AI virtual companion products.	
Perceived enjoyment	I find interacting with AI virtual companion products enjoyable.	Jo, 2022; Yuan et al., 2022
	I find interacting with AI virtual companion products relaxing.	
	I can become fully immersed in the services provided by AI virtual companion products.	
Perceived anthropomorphism	I believe that AI virtual companion products appear to have self-awareness.	Yuan et al., 2022; Zhou and Zhang, 2024
	I believe that the tone and behavior of AI virtual companion products make me feel as if I am communicating with a real person.	
	I believe that AI virtual companion products seem to possess a sense of life.	
Social anxiety	In general, I am a shy person.	Jin and Youn, 2021; Lavie and Tractinsky, 2004; Yuan et al., 2022
	I tend to avoid talking to people I do not know.	
	I often feel nervous when talking to people I am not familiar with.	
Behavioral intention	I am willing to continue using AI virtual companion products in the future.	Saleh et al., 2022; Xie et al., 2025
	I am willing to recommend AI virtual companion products to my friends or family.	
	I intend to increase my frequency of using AI virtual companion products in the future.	

## 4 Results

### 4.1 Descriptive statistical analysis

The basic demographic information of the sample is presented in Table 2. A total of 712 valid responses were collected in this study, including 367 males (51.5%) and 345 females (48.5%), indicating a relatively balanced gender distribution. The age structure of respondents was skewed toward younger groups, with individuals aged 18–35 accounting for 65.9%, and those aged 36–55 representing 34.1%. Regarding educational background, 400 participants (56.2%) held a bachelor's or associate degree, while 122 participants (17.1%) had a graduate degree or above.

**Table 2:** Basic demographic information.

Characteristics	Categories	*N*	%
Gender	Male	367	51.5
	Female	345	48.5
Age group	18~25	266	37.4
	26~35	203	28.5
	36~45	134	18.8
	46~55	109	15.3
Education	High school or below/ Vocational secondary school	190	26.7
	Bachelor's degree or associate degree	400	56.2
	Master's or doctoral degree	122	17.1

### 4.2 Common method bias test

Because all variables in this study were measured using self-reported questionnaire data collected at a single time point, it was necessary to test for common method bias. First, Harman's single-factor test was conducted by entering all measurement items into an unrotated exploratory factor analysis. The results showed that the first factor accounted for 33.549% of the total variance, which is below the commonly used threshold of 50%, indicating that no serious common method bias was evident. In addition, VIF collinearity diagnostics were performed for the predictor variables in the model. The results showed that the VIF values of the predictor variables ranged from 1.029 to 1.558, all below the recommended threshold of 3.3. Taken together, these statistical results suggest that common method bias was unlikely to have seriously affected the conclusions of this study.

### 4.3 SEM-based analysis of usage intention toward AI virtual companion products

#### 4.3.1 Measurement model testing

To evaluate the goodness of fit between the theoretical model and the collected data, this study employed confirmatory factor analysis (CFA) to assess the reliability and validity of the measurement model.

For reliability testing, indicator reliability was first assessed based on factor loadings. As shown in Table 3, the factor loadings of all observed variables ranged from 0.863 to 0.906, exceeding the threshold of 0.7, indicating good indicator reliability for each measurement item (Moussaïd et al., 2013; Sobti, 2019). Internal consistency reliability was further examined using composite reliability (CR) and Cronbach's α. Results showed that Cronbach's α values ranged from 0.855 to 0.869 and CR values ranged from 0.912 to 0.919, all significantly higher than the recommended cutoff of 0.7, demonstrating that the scale has high reliability (Izah et al., 2023).

**Table 3:** Reliability and convergent validity of the measurement model.

Constructs	Items	Factor loadings	Cronbach's α	CR	AVE
Perceived usefulness	PU1	0.863	0.856	0.912	0.775
	PU2	0.879			
	PU3	0.899			
Perceived ease of use	PEU1	0.890	0.861	0.915	0.783
	PEU2	0.883			
	PEU3	0.881			
Perceived enjoyment	PE1	0.889	0.855	0.912	0.775
	PE2	0.873			
	PE3	0.879			
AI trust	AIT1	0.883	0.865	0.917	0.787
	AIT2	0.891			
	AIT3	0.887			
Perceived anthropomorphism	PA1	0.895	0.869	0.919	0.791
	PA2	0.891			
	PA3	0.883			
Social anxiety	SA1	0.906	0.863	0.916	0.784
	SA2	0.869			
	SA3	0.882			
Behavioral intention	BI1	0.888	0.869	0.919	0.792
	BI2	0.883			
	BI3	0.899			

For validity testing, convergent validity was assessed using the average variance extracted (AVE). As shown in Table 3, the AVE values of all latent variables ranged from 0.775 to 0.792, well above the recommended value of 0.5, confirming good convergent validity of the model (Cheung et al., 2024). With regard to discriminant validity, the results in Table 4 show that the loading of each item on its intended construct was higher than its cross-loadings on other constructs. In addition, the Fornell-Larcker test results in Table 5 show that the square root of the AVE for each construct was greater than its correlations with other constructs, indicating that the measurement model had good discriminant validity.

**Table 4:** Reliability and validity measures.

Constructs	PU	PEU	PE	AIT	SA	PA	BI
PU1	**0.863**	0.440	0.283	0.351	0.360	0.060	0.302
PU2	**0.879**	0.440	0.316	0.382	0.401	0.073	0.338
PU3	**0.899**	0.410	0.276	0.374	0.398	0.124	0.385
PEU1	0.435	**0.890**	0.379	0.333	0.360	0.114	0.316
PEU2	0.442	**0.883**	0.336	0.307	0.409	0.054	0.285
PEU3	0.414	**0.881**	0.370	0.338	0.377	0.103	0.303
PE1	0.313	0.359	**0.889**	0.403	0.207	0.091	0.391
PE2	0.280	0.369	**0.873**	0.329	0.219	0.079	0.380
PE3	0.278	0.355	**0.879**	0.326	0.218	0.106	0.376
AIT1	0.370	0.347	0.376	**0.883**	0.287	0.103	0.422
AIT2	0.372	0.322	0.339	**0.891**	0.292	0.132	0.412
AIT3	0.375	0.314	0.352	**0.887**	0.308	0.150	0.428
SA1	0.427	0.400	0.229	0.309	**0.906**	0.101	0.304
SA2	0.368	0.366	0.211	0.280	**0.869**	0.097	0.256
SA3	0.368	0.376	0.207	0.296	**0.882**	0.103	0.256
PA1	0.087	0.078	0.087	0.129	0.117	**0.895**	0.257
PA2	0.089	0.107	0.106	0.133	0.097	**0.891**	0.229
PA3	0.091	0.092	0.086	0.125	0.087	**0.883**	0.220
BI1	0.321	0.287	0.382	0.405	0.273	0.241	**0.888**
BI2	0.355	0.306	0.375	0.420	0.267	0.244	**0.883**
BI3	0.368	0.317	0.401	0.440	0.285	0.225	**0.899**

Bold values indicate the loadings of each item on its corresponding construct.

**Table 5:** Discriminant validity.

Constructs	PU	PEU	PE	AIT	SA	PA	BI
PU	**0.880**						
PEU	0.486	**0.885**					
PE	0.330	0.410	**0.880**				
AIT	0.420	0.369	0.401	**0.887**			
SA	0.440	0.431	0.244	0.145	**0.886**		
PA	0.100	0.103	0.104	0.334	0.113	**0.890**	
BI	0.391	0.341	0.434	0.474	0.309	0.266	**0.890**

The bold diagonal values represent the square root of AVE, and the lower triangle shows the Pearson correlations.

#### 4.3.2 Structural model testing

**Figure 2:**
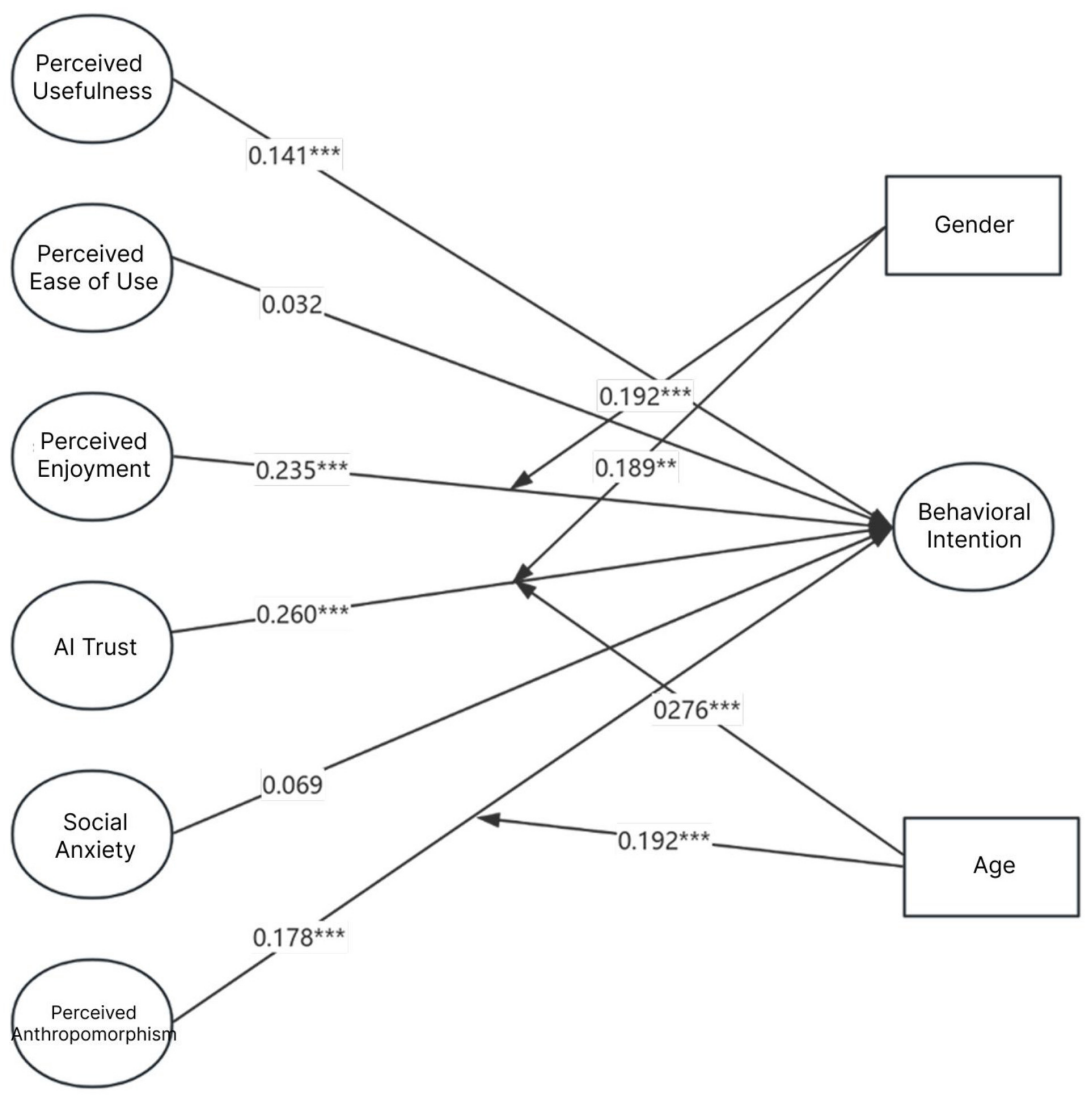
Structural model results. ***p < 0.01.

This study employed the bootstrapping method in Smart PLS to test the structural model, with 5,000 resamples and a 95% confidence interval. The path coefficients (β), *T*-statistics, significance levels (*P*), and hypothesis testing results for the relationships among latent variables are presented in Table 6. The structural model results are illustrated in Figure 2.

**Table 6:** Hypothesis testing results of the structural model.

Hypothesis	Path	β	Standard deviation	T statistics	*P* values	Supported
H1	PU → BI	0.141	0.038	3.711	0.000	Yes
H2	PEU → BI	0.032	0.038	0.844	0.399	No
H3	PE → BI	0.235	0.036	6.590	0.000	Yes
H4	AIT → BI	0.260	0.037	7.000	0.000	Yes
H5	PA → BI	0.178	0.030	6.017	0.000	Yes
H6	SA → BI	0.069	0.037	1.869	0.062	No

β represents the standardized path coefficient; P < 0.05 indicates statistical significance.

First, perceived usefulness, perceived enjoyment, AI trust, and perceived anthropomorphism were all significantly and positively associated with users' usage intention. Among them, AI trust showed the strongest association (β = 0.260, *P* < 0.001), followed by perceived enjoyment (β = 0.235, *P* < 0.001), perceived anthropomorphism (β = 0.178, *P* < 0.001), and perceived usefulness (β = 0.141, *P* < 0.001). Second, the direct associations of perceived ease of use (β = 0.032, *P* = 0.399) and social anxiety (β = 0.069, *P* = 0.062) with usage intention did not reach statistical significance.

To evaluate the explanatory power and predictive capability of the model, this study further calculated the coefficient of determination (*R*^2^) and the predictive relevance index (*Q*^2^), which measure the overall explanatory effect of exogenous variables on endogenous variables. As shown in Table 7, the model explains 36.0% of the variance in usage intention. According to the criteria proposed by Hair et al. (2011), an *R*^2^ value greater than 0.25 indicates acceptable explanatory power, greater than 0.50 indicates moderate explanatory power, and greater than 0.75 indicates substantial explanatory power. Therefore, the explanatory effect of this model on usage intention reaches an acceptable level. In addition, the *Q*^2^ value for usage intention was 0.348, which is close to the 0.35 threshold for strong predictive relevance (Hair et al., 2019). This result indicates that the model not only demonstrates significant predictive relevance but also possesses relatively strong predictive capability.

**Table 7:** *R*^2^ and *Q*^2^ values of endogenous constructs.

Construct	*R* ^2^	*R*^2^(adjusted)	*Q*^2^predict
Behavioral intention	0.360	0.355	0.348

#### 4.3.3 Moderation effect testing

To examine the moderating effects of gender and age, this study first conducted group segmentation of the sample. Specifically, in terms of gender, the sample was divided into male (*n* = 367) and female (*n* = 345) groups; in terms of age, it was divided into an older group (36–55 years, n = 243) and a younger group (18–35 years, *n* = 469). Subsequently, the Partial Least Squares Multi-Group Analysis (PLS-MGA) method in SmartPLS was employed for testing (Wang et al., 2019).

As shown in Tables 8, 9, the results are as follows: First, regarding gender, the effects of AI trust (β = 0.349, *P* < 0.001) and perceived enjoyment (β = 0.317, *P* < 0.001) on usage intention were significantly stronger for females than for males.

**Table 8:** Moderation effect testing (gender).

Path	Path coefficient		Difference in path coefficients (Δβ)
	Female	Male	
AIT → BI	0.349^***^	0.160^***^	0.189^**^
PE → BI	0.317^***^	0.125^**^	0.192^***^

^***^p < 0.01, ^**^p < 0.05; Δβ indicates the difference in path coefficients between the two groups.

**Table 9:** Moderation effect testing (age).

Path	Path coefficient		Difference in path coefficients (Δβ)
	Younger group	Older group	
AIT → BI	0.152^***^	0.428^***^	0.276^***^
PA → BI	0.096^**^	0.288^***^	0.192^***^

^***^p < 0.01, ^**^p < 0.05; Δβ indicates the difference in path coefficients between the two groups.

Second, regarding age, the effects of AI trust (β = 0.428, *P* < 0.001) and perceived anthropomorphism (β = 0.288, *P* < 0.001) on usage intention were significantly stronger for the older group than for the younger group.

### 4.4 SEM-ANN-based analysis of the usage intention model for AI virtual companion products

**Figure 3:**
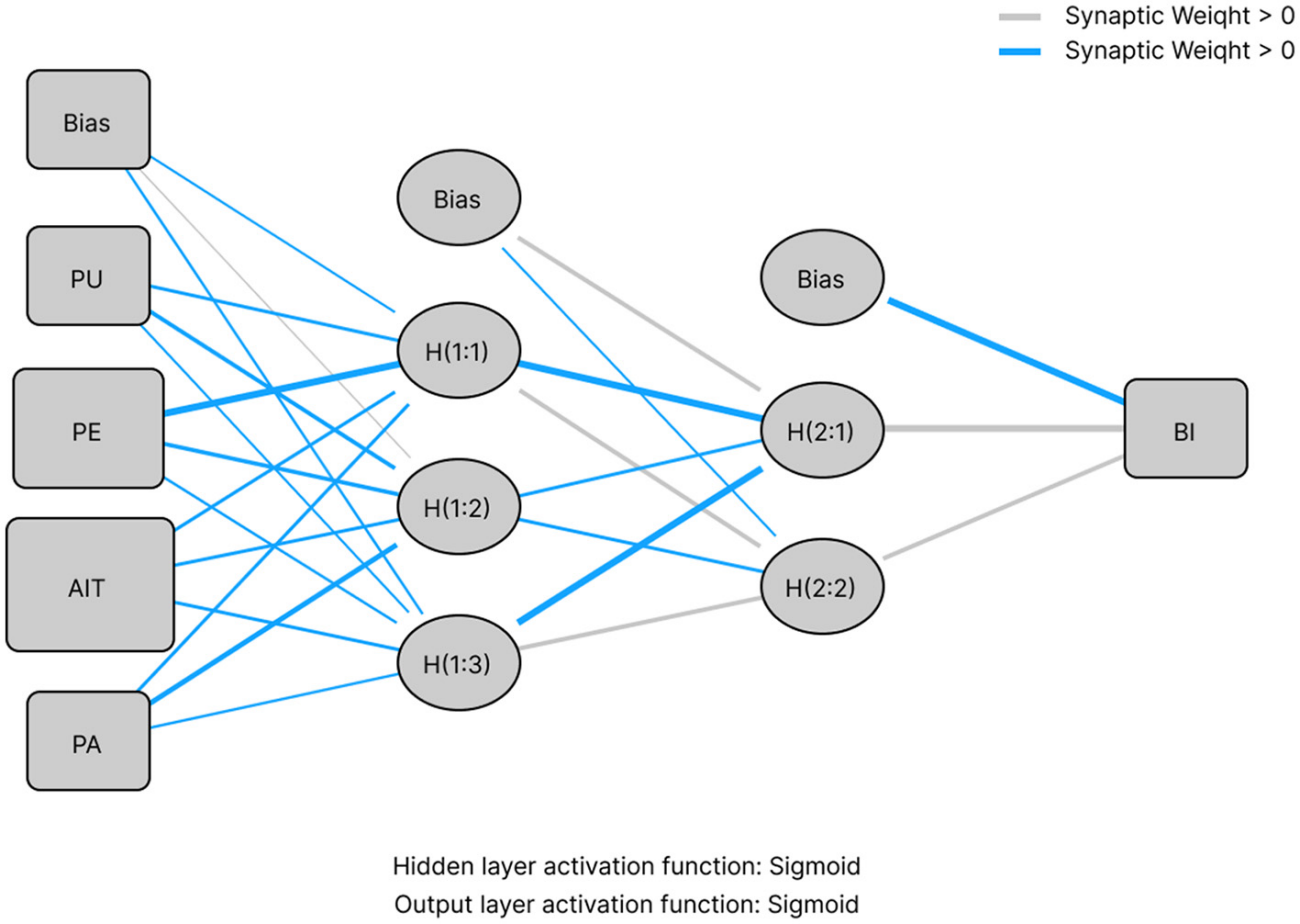
Feedforward neural network model.

To further examine the relative contribution of significant predictors to usage intention within a nonlinear predictive framework, this study introduced an artificial neural network (ANN) approach based on the results of the PLS-SEM analysis and constructed a hybrid SEM-ANN analytical model. Drawing on the approach of Liébana-Cabanillas et al. (2017) and taking into account the path testing results of PLS-SEM and the predictive characteristics of ANN, this study included only the four variables that reached statistical significance in the PLS-SEM analysis in the ANN input layer: perceived usefulness (PU), perceived enjoyment (PE), AI trust (AIT), and perceived anthropomorphism (PA). Perceived ease of use (PEU) and social anxiety (SA) were not included in the ANN model because they did not reach statistical significance in the PLS-SEM path analysis, thereby avoiding potential noise from non-significant variables in the predictive model. The output-layer variable of the ANN model was usage intention (BI), and the specific structure is shown in Figure 3.

In terms of model structure, this study adopted a multilayer perceptron (MLP) neural network model. The model included an input layer, a hidden layer, and an output layer. The input layer contained four neurons corresponding to PU, PE, AIT, and PA, respectively, while the output layer contained one neuron corresponding to BI. The hidden-layer structure was automatically determined by the SPSS neural network module based on the training data. Sigmoid activation functions were used in both the hidden layer and the output layer. To improve the stability of model training and reduce the influence of scale differences on the training results, all input and output variables were normalized to the [0,1] range. To reduce instability caused by a single sample split and to control the risk of overfitting, this study adopted a ten-fold cross-validation approach. Specifically, in each round of model training, approximately 90% of the sample was used for training and approximately 10% was used for testing, and a total of 10 ANN sub-models were constructed. Model predictive accuracy was evaluated using the root mean square error (RMSE) (Chong et al., 2015; Liébana-Cabanillas et al., 2017; Ooi and Tan, 2016). The training and testing results are presented in Table 10. The mean RMSE was 0.033 for the training set and 0.093 for the testing set, and the standard deviation of the testing-set RMSE was low, indicating that the model showed relatively stable predictive performance across different data splits and did not exhibit obvious overfitting (Dang et al., 2023; Wong et al., 2022).

**Table 10:** Predictive accuracy of the ANN model.

Neural Network	Training		Testing	
	*N*	RMSE	*N*	RMSE
ANN1	635	0.034	77	0.093
ANN2	632	0.031	80	0.091
ANN3	628	0.035	84	0.088
ANN4	636	0.037	76	0.094
ANN5	643	0.036	69	0.097
ANN6	639	0.034	73	0.097
ANN7	639	0.032	73	0.096
ANN8	634	0.031	78	0.091
ANN9	634	0.032	78	0.092
ANN10	639	0.031	73	0.096
Mean	0.033		0.093	
SD	0.002		0.003	

In addition, to further assess the relative predictive contribution of each input variable in the ANN model, this study conducted a sensitivity analysis, and the results are shown in Table 11. The relative importance values obtained from the sensitivity analysis reflect the contribution of each input variable to the prediction of the output variable BI. To facilitate comparison across variables, the highest relative importance value was standardized to 100%, and the normalized relative importance of the remaining variables was calculated accordingly. According to the results shown in Table 12, AI trust had the highest normalized relative importance in the ANN model, followed by perceived anthropomorphism, perceived enjoyment, and perceived usefulness. This ranking differs to some extent from the ordering of path coefficients in the PLS-SEM results, indicating that ANN can provide supplementary information from a nonlinear predictive perspective that differs from linear path analysis.

**Table 11:** Results of sensitivity analysis.

ANN	PU	PE	AIT	PA
ANN1	0.175	0.287	0.350	0.189
ANN2	0.243	0.273	0.309	0.176
ANN3	0.265	0.224	0.345	0.165
ANN4	0.268	0.232	0.407	0.902
ANN5	0.243	0.274	0.324	0.158
ANN6	0.270	0.370	0.273	0.807
ANN7	0.253	0.215	0.325	0.206
ANN8	0.203	0.290	0.311	0.196
ANN9	0.148	0.305	0.325	0.222
ANN10	0.248	0.292	0.315	0.145
Average relative importance	0.232	0.276	0.328	0.317
Normalized relative importance (%)	70.7%	84.1%	100%	99.7%

**Table 12:** Comparison of PLS-SEM and ANN results.

Path	Path coefficient (PLS-SEM)	Normalized relative importance (%) (ANN)	Ranking based on PLS-SEM	Ranking based on ANN
PU → BI	0.141	70.7%	4	4
PE → BI	0.235	84.1%	2	3
AIT → BI	0.260	100%	1	1
PA → BI	0.178	99.7%	3	2

Furthermore, to evaluate the predictive capability of the ANN model, a sensitivity analysis was conducted, and the results are presented in Table 11. Based on the normalized importance indices shown in Table 12, perceived anthropomorphism demonstrated a higher importance level than perceived enjoyment within the model. This finding differs from the results of the SEM-based analysis, suggesting that ANN provides additional insights into the relative influence of key factors in predicting users' intention to use AI virtual companion products.

## 5 Discussion

### 5.1 Effects of the extended TAM3 model variables on the usage intention of AI virtual companion products

First, perceived usefulness was significantly and positively associated with usage intention. This indicates that the more users believe AI virtual companions can provide emotional support, daily companionship, and interactive experiences, the stronger their willingness to use them. This finding supports the view of Merrill Jr et al. (2022), emphasizing the critical role of perceived usefulness in enhancing user acceptance. Therefore, product design should further strengthen the emotional interaction, psychological support, and personalized service functions of AI virtual companion products to enhance users perceived practical value.

Second, the direct relationship between perceived ease of use and usage intention was not significant. This result differs from the findings reported by Sultan Hammad Alshammari and colleagues (Alshammari and Babu, 2025). One possible explanation is that the sample in this study consisted mainly of younger users, who are generally familiar with smartphones and interactive applications and are therefore less sensitive to basic operational difficulty. In addition, most AI virtual companion products adopt low-threshold interaction formats such as chat windows, virtual character interaction, or voice communication, allowing users to complete basic use without complex learning. Therefore, in the context of AI virtual companion products, perceived ease of use may function more as a basic condition: it may be a prerequisite for users' willingness to try the product, but it may not necessarily constitute a core differentiating factor for continued usage intention. By contrast, users may pay greater attention to whether the product can provide trustworthy responses, stable emotional companionship, natural interaction experiences, and continuity in personalized relationships. Accordingly, future development of AI virtual companion products should not remain limited to lowering operational barriers, but should further improve the quality of emotional interaction, long-term memory capabilities, privacy and security protection, and trust-building mechanisms.

Third, perceived enjoyment was significantly and positively associated with usage intention. This suggests that the entertainment and emotional satisfaction provided by AI virtual companion products serve as key motivators for continuous use. This finding aligns with the conclusions of Jo (2022), who emphasized the importance of perceived enjoyment in shaping user acceptance. Accordingly, product development should prioritize creating engaging interface designs, diverse interaction modes, and personalized recommendation features to enhance users' long-term immersion and enjoyment experiences.

### 5.2 Effects of AI-related variables on usage intention

The results of this study show that AI trust was the variable most strongly associated with usage intention. This finding suggests that, in the context of AI virtual companion products, user acceptance is not based solely on functional efficiency or entertainment experience, but also depends heavily on users' judgments regarding system reliability, safety, privacy protection, and interaction boundaries. Unlike general tool-oriented AI products, AI virtual companions typically involve a higher degree of emotional expression, personalized dialogue, relationship memory, and private information exchange. During use, users may express emotional distress, intimate needs, or personal experiences to the system. Therefore, they first need to determine whether the system is stable, trustworthy, capable of protecting their data, and unlikely to misuse their emotional investment. In this sense, AI trust is not only a prerequisite for technology acceptance, but also an important psychological foundation for users' willingness to establish sustained interactive relationships with AI virtual companions. This finding supports the view of Hyesun Choung and colleagues regarding the critical role of AI trust in enhancing user acceptance (Choung et al., 2023).

At the same time, perceived anthropomorphism was also significantly and positively associated with usage intention. The results indicate that the more users perceive human-like characteristics in the language style, interaction mode, emotional responses, and virtual image of AI virtual companions, the more likely they are to develop immersion, a sense of closeness, and willingness to continue interacting. This finding is consistent with the view of Sara H. Hsieh and colleagues that anthropomorphic features can enhance user acceptance and continuance intention (Hsieh and Lee, 2021). It should be noted that perceived anthropomorphism in this study mainly refers to users' human-like perceptions formed on the basis of the language, behavior, and interaction style of AI virtual companions, rather than an objective judgment about whether AI truly possesses consciousness, life, or emotional capacity. However, anthropomorphic design should also be combined with transparency cues and boundary explanations, in order to avoid users mistakenly believing that AI has genuine emotions, autonomous consciousness, or a status equivalent to real human relationships because of highly anthropomorphic interaction experiences.

Overall, this study verifies the central roles of AI trust and perceived anthropomorphism in influencing users' intention to use virtual companion products. These two factors correspond to the “security-risk” and “emotional-immersion” dimensions, respectively. Building trust is the prerequisite for long-term acceptance, while enhancing anthropomorphism serves as the core driver of emotional engagement. In practice, the development of AI virtual companions should strike a balance between technical security and human-centered experience: strengthening data protection and transparency on one hand, while improving anthropomorphic design and natural interaction on the other, thereby enhancing users' usage intention from both rational and emotional perspectives.

### 5.3 Effects of social and psychological factors on usage intention

The results of this study show that the direct relationship between social anxiety and usage intention was not significant. This suggests that social anxiety itself may not be a direct factor driving users to adopt AI virtual companion products. Although AI virtual companion products may provide a low-pressure communication environment to some extent and may help relieve loneliness, anxiety, or real-world social pressure, such effects do not necessarily translate directly into overall usage intention. In other words, social anxiety may not be a proximal direct predictor, but may instead indirectly influence users' acceptance of AI virtual companions through other psychological mechanisms (Xie and Wang, 2024).

This explanation is also consistent with prior discussions regarding the mechanisms through which social anxiety operates. For individuals with higher levels of social anxiety, the motivation to use AI virtual companions may not stem from “social anxiety” itself, but rather from loneliness, interpersonal avoidance, self-disclosure needs, or parasocial relationship tendencies that are further triggered by social anxiety. For example, users with higher social anxiety may be more inclined to choose AI interaction environments with lower risk and less evaluative pressure because of the stress associated with real interpersonal interaction. They may also seek alternative companionship through AI virtual companions because of stronger needs for emotional expression and self-disclosure. Therefore, social anxiety is more likely to be indirectly associated with usage intention through mediating mechanisms such as loneliness, interpersonal avoidance, self-disclosure needs, or parasocial relationship formation.

Because this study did not measure these mediating variables, the indirect path of social anxiety was not further tested in the current model. This result suggests that future research should not focus only on the direct relationship between social anxiety and usage intention, but should further develop mediation or serial mediation models to examine whether social anxiety influences the intention to use AI virtual companions through more proximal psychological mechanisms such as loneliness, interpersonal avoidance, self-disclosure needs, or parasocial relationship formation.

### 5.4 Moderating effects of user characteristics

The results indicate that both gender and age exhibit significant moderating effects in the model. Specifically, female users' usage intention is more strongly influenced by AI trust and perceived enjoyment, suggesting that they pay greater attention to the reliability, security, and emotional experience of AI virtual companions. Regarding age, older users demonstrate higher dependence on AI trust and anthropomorphic features, indicating that they value system stability, safety, and natural human-like interaction. This finding is consistent with prior research suggesting that gender and age significantly influence users' psychological perceptions and behavioral pathways, underscoring the importance of demographic differences in technology acceptance studies (Jo, 2022; Zhang et al., 2023).

However, this study also differs from some prior findings that proposed younger users are more receptive to anthropomorphic features and male users exhibit higher AI trust levels (Kozak and Fel, 2024; Symasek et al., 2025). These discrepancies may arise from differences in sample characteristics, technological maturity, and sociocultural contexts—all of which jointly influence users' perceptions of trust and anthropomorphism. Therefore, this study not only provides a reasonable explanation for inconsistencies in existing literature but also offers a new perspective for future research exploring the interaction among demographic characteristics, technological environments, and cultural factors.

Practically, the design and promotion of virtual companion products should follow differentiated and stratified strategies. For female users, developers should emphasize emotional care and security features to enhance trust and emotional satisfaction; for older users, the design should highlight stability, reliability, and long-term companionship to fulfill their need for security and continuous interaction. Developers should therefore create diverse functional modules to meet the specific needs of various user groups, enabling more targeted design and precise market segmentation.

### 5.5 Model interpretation and predictive capability

To assess the explanatory and predictive power of the research model, both SEM and ANN methods were applied. The SEM results showed that perceived usefulness, perceived enjoyment, AI trust, and perceived anthropomorphism were all significantly and positively associated with usage intention. The model explained 36.0% of the variance in usage intention, which represents an acceptable to moderate level. In addition, the *Q*^2^ value of 0.348 approached the strong predictive relevance threshold of 0.35, indicating that the model possessed both significant predictive relevance and strong predictive capability. Furthermore, the ANN results revealed mean Root Mean Square Errors (RMSEs) of 0.033 for the training set and 0.093 for the testing set, demonstrating that the ANN model achieved good predictive accuracy and higher explanatory capacity.

In terms of variable importance rankings, there were some differences between the SEM and ANN results. Based on the SEM path coefficients, AI trust showed the strongest association with usage intention, followed by perceived enjoyment, perceived anthropomorphism, and perceived usefulness. However, the normalized relative importance results from the ANN analysis showed that AI trust remained the most important predictor, but perceived anthropomorphism had higher relative importance than perceived enjoyment, while perceived usefulness still ranked last. Thus, the two methods were consistent in the rankings of AI trust and perceived usefulness, but differed in the middle ordering of perceived enjoyment and perceived anthropomorphism.

This difference does not mean that the results of the two methods are contradictory; rather, it reflects differences in their analytical logic and evaluation indicators. SEM path coefficients mainly capture the strength of linear associations among variables in the theoretical model and emphasize path direction and significance testing, whereas the normalized relative importance derived from ANN reflects the relative contribution of input variables to the output variable within a nonlinear predictive framework and can capture both linear and nonlinear relationships among variables (Sohaib et al., 2019). Therefore, the fact that perceived enjoyment had a higher path coefficient than perceived anthropomorphism in SEM, while perceived anthropomorphism had greater predictive importance than perceived enjoyment in ANN, may suggest that perceived anthropomorphism plays a more complex predictive role in shaping intention to use AI virtual companions. It also highlights the complementary value of ANN relative to linear methods such as PLS-SEM (Wang et al., 2022). In other words, users' anthropomorphic perceptions of AI virtual companions may not be associated with usage intention in a simple linear manner, but may instead operate jointly with factors such as trust, immersion, and emotional relationship building.

Overall, the integration of SEM and ANN provides a complementary perspective for understanding intention to use AI virtual companion products. SEM helps test the linear association paths in the theoretical model, whereas ANN further reveals the relative importance of significant variables within a nonlinear predictive model. Therefore, the two-stage SEM-ANN analysis not only strengthens the model's predictive explanation, but also offers a research pathway that combines theory testing with predictive analysis for future studies (Bellary et al., 2024).

### 5.6 Practical implications

Based on the above findings, this study offers the following practical implications for the design of AI virtual companion products and user engagement strategies. First, because AI trust was the most important predictor, developers should prioritize the establishment of clear and perceptible trust mechanisms. For example, platforms can provide transparent explanations of data use, privacy permission settings, conversation record management, memory deletion functions, and safety reminder mechanisms, so that users can clearly understand how their personal data are collected, stored, and used.

Second, in terms of emotional experience design, developers should focus on improving relational continuity and response quality in long-term interaction. For example, they can optimize contextual memory, emotion recognition, personalized forms of address, feedback on relationship progression, and emotional support strategies, so that users can experience companionship that feels stable, continuous, and responsive. At the same time, anthropomorphic design should remain moderate. While it should enhance natural communication and immersive experience, it should also clearly explain the functional scope and interaction boundaries of AI, so as to avoid users mistakenly believing that AI possesses genuine emotions or autonomous consciousness because of highly anthropomorphic interaction experiences, and to reduce the possibility that excessive human-like cues may trigger unrealistic emotional dependence.

Third, adjustable and layered interaction modes should be provided for different user groups. For users who value safety and emotional experience, functions related to privacy protection, emotional care, and reliability cues can be strengthened. For users who seek entertainment and immersion, character settings, interactive storylines, voice expression, and personalized recommendations can be enhanced. For users who may experience social difficulties or loneliness, low-pressure spaces for expression, emotional diaries, companionship reminders, and self-disclosure support functions may be designed, but the product should not be positioned simply as a substitute for real-world social relationships. Through these more specific design strategies, AI virtual companion products can achieve a more reasonable balance among trust, safety, emotional experience, and user engagement.

## 6 Conclusion and future directions

Based on an extended TAM3 model and AI-related feature variables, this study constructed a theoretical model of intention to use AI virtual companion products and adopted a hybrid method combining SEM and ANN for empirical analysis, in order to systematically examine the key factors associated with users' acceptance of AI virtual companion products and their relative predictive roles. The SEM results showed that perceived usefulness, perceived enjoyment, AI trust, and perceived anthropomorphism were significantly and positively associated with usage intention, whereas the direct associations of perceived ease of use and social anxiety with usage intention were not significant. Gender and age also showed moderating effects on some of the paths. Further ANN analysis showed that AI trust remained the most important predictor in terms of relative importance, while perceived usefulness ranked lowest. However, unlike the SEM path coefficients, the ANN results showed that perceived anthropomorphism had higher relative importance than perceived enjoyment, indicating that SEM and ANN were consistent in identifying key predictors while also providing complementary information.

The contributions of this study are mainly reflected in three aspects. First, at the theoretical level, this study does not merely combine existing variables, but instead contextualizes traditional technology acceptance variables in TAM3 together with variables such as AI trust, perceived anthropomorphism, and social anxiety, based on the unique characteristics of AI virtual companions as emotionally relational AI products. In doing so, it extends the explanatory framework for intention to use AI virtual companion products. Second, at the methodological level, this study combines SEM and ANN, thereby not only testing the linear association paths in the theoretical model, but also further comparing the relative importance of different variables within a nonlinear predictive framework, thus providing a complementary analytical perspective for understanding users' intention to use AI virtual companion products. Third, at the practical level, the results suggest that the optimization of AI virtual companion products should not focus only on functional usability or operational convenience, but should also pay attention to trust mechanisms, privacy protection, the appropriate degree of anthropomorphic interaction, and the differentiated participation needs of different user groups.

Although this study offers a certain degree of theoretical and methodological innovation, it still has several limitations. First, the sample mainly consisted of young users and was drawn only from Chinese users, which may affect the cross-cultural generalizability of the findings. Second, this study examined AI virtual companions as an overall product category rather than focusing on any specific platform or application. Although the questionnaire provided a unified explanation of “AI virtual companion products” before the formal items, listed common products such as X EVA as examples, and required respondents to have used the above products or products with similar functions before continuing, the study did not further record the specific product types used by each respondent, their usage frequency, or their duration of use. Therefore, the findings are better understood as reflecting the overall acceptance tendency of users with relevant experience toward the category of AI virtual companion products, rather than acceptance of any specific platform. Third, the data were cross-sectional and therefore could not capture dynamic changes in users over the course of long-term interaction. Accordingly, the present findings are more appropriately interpreted as associations or predictive relationships among variables rather than strict causal mechanisms. Fourth, the measurement of variables relied on self-reported questionnaire data collected at a single time point. Although this study tested for common method bias using Harman's single-factor test and VIF collinearity diagnostics, and the results suggested that common method bias was unlikely to have seriously affected the conclusions, single-source data may still involve some degree of self-report bias and common method bias. Fifth, this study tested only the direct relationship between social anxiety and usage intention and did not further incorporate possible mediating variables such as loneliness, interpersonal avoidance, self-disclosure needs, or parasocial relationship formation. Therefore, the study cannot fully explain whether social anxiety is indirectly associated with intention to use AI virtual companion products through more proximal psychological mechanisms. Sixth, although the perceived anthropomorphism items in this study were adapted from existing anthropomorphism scales used in AI-related contexts, this construct in the context of AI virtual companions may involve different layers of human-like perception, and its conceptual boundaries still require further refinement. Finally, although the integration of SEM and ANN improved predictive accuracy, there is still room for improvement in explaining complex variables such as emotion and psychology.

Future research may proceed in several directions. First, future studies should further expand the sample to include users from different age groups, occupations, and cultural backgrounds, especially cross-national and cross-cultural samples, in order to improve the external validity of the findings. Second, future studies may adopt longitudinal or experimental designs to examine changes in user acceptance across different stages of use and under long-term interaction, and to further test potential causal relationships between relevant variables and usage intention. Third, future research may further develop mediation or serial mediation models to examine whether social anxiety is indirectly associated with users' intention to use AI virtual companion products through psychological mechanisms such as loneliness, interpersonal avoidance, self-disclosure needs, or parasocial relationship formation. Fourth, future studies may incorporate cross-cultural comparisons to explore the influence of sociocultural differences on the acceptance of virtual companion products. Fifth, future research may further distinguish different dimensions of perceived anthropomorphism, such as appearance anthropomorphism, interaction anthropomorphism, psychological-attribute anthropomorphism, and consciousness attribution, in order to test whether different types of human-like perception show differentiated associations with intention to use AI virtual companion products and to improve the measurement precision of the related construct. Sixth, future studies may incorporate multi-source data, such as behavioral logs, interview materials, platform usage records, physiological signals, or longitudinal tracking data, in order to reduce the possible bias introduced by single-source self-reported data and to enhance the objectivity and multidimensional validation of the findings.

## Data Availability

The raw data supporting the conclusions of this article will be made available by the authors, without undue reservation.
